# Liquid and Vapor Phase of Four Conifer-Derived Essential Oils: Comparison of Chemical Compositions and Antimicrobial and Antioxidant Properties

**DOI:** 10.3390/ph14020134

**Published:** 2021-02-08

**Authors:** Stefania Garzoli, Valentina Laghezza Masci, Valentina Caradonna, Antonio Tiezzi, Pierluigi Giacomello, Elisa Ovidi

**Affiliations:** 1 Department of Drug Chemistry and Technology, Sapienza University, 00185 Rome, Italy; pierluigi.giacomello@uniroma1.it; 2 Department for the Innovation in Biological, Agrofood and Forestal Systems, Tuscia University, 01100 Viterbo, Italy; laghezzamasci@unitus.it (V.L.M.); valecarad@gmail.com (V.C.); antoniot@unitus.it (A.T.); eovidi@unitus.it (E.O.)

**Keywords:** *Pinus cembra* L., *Pinus mugo* Turra, *Picea abies* L., *Abies alba* M., essential oil, chemical investigation, HS-GC/MS, antibacterial activity, antioxidant activity

## Abstract

In this study, the chemical composition of the vapor and liquid phase of *Pinus cembra* L., *Pinus mugo* Turra, *Picea abies* L., and *Abies Alba* M. needles essential oils (EOs) was investigated by Headspace-Gas Chromatography/Mass Spectrometry (HS-GC/MS). In the examined EOs, a total of twenty-eight components were identified, most of which belong to the monoterpenes family. α-Pinene (16.6–44.0%), β-pinene (7.5–44.7%), limonene (9.5–32.5%), and γ-terpinene (0.3–19.7%) were the most abundant components of the liquid phase. Such major compounds were also detected in the vapor phase of all EOs, and α-pinene reached higher relative percentages than in the liquid phase. Then, both the liquid and vapor phases were evaluated in terms of antibacterial activity against three Gram-negative bacteria (*Escherichia coli*, *Pseudomonas fluorescens*, and *Acinetobacter bohemicus*) and two Gram-positive bacteria (*Kocuria marina* and *Bacillus cereus*) using a microwell dilution assay, disc diffusion assay, and vapor phase test. The lowest Minimum Inhibitory Concentration (MIC) (13.28 mg/mL) and Minimal Bactericidal Concentration (MBC) (26.56 mg/mL) values, which correspond to the highest antibacterial activities, were reported for *P. abies* EO against *A. bohemicus* and for *A. alba* EO against *A. bohemicus* and *B. cereus*. The vapor phase of all the tested EOs was more active than liquid phase, showing the inhibition halos from 41.00 ± 10.15 mm to 80.00 ± 0.00 mm for three bacterial strains (*A. bohemicus*, *K. marina*, and *B. cereus*). Furthermore, antioxidant activities were also investigated by 2,2-diphenyl-1-picrylhydrazyl (DPPH) and 2,2′-azinobis (3- ethylbenzothiazoline-6-sulfonic acid) diammonium salt (ABTS) assays, and a concentration-dependent antioxidant capacity for all EOs was found. *P. mugo* EO showed the best antioxidant activity than the other Pinaceae EOs. The four Pinaceae EOs could be further investigated for their promising antibacterial and antioxidant properties, and, in particular, α-pinene seems to have interesting possibilities for use as a novel natural antibacterial agent.

## 1. Introduction

Since ancient times, plants are a source of different kinds of compounds that humans used for their numerous biological activities and as a source for drug development [1]. Nowadays, the studies on antioxidant and antimicrobial activities of natural products are of considerable interest due to the importance of identifying and characterizing new bioactive molecules for applications in different fields as food preservation and packaging, antibiotically resistance phenomenon, and plant diseases.

Among plant secondary metabolites, essential oils (EOs), biosynthesized by glandular trichomes and other secretory structures in plants, are liquids particularly rich in volatile molecules such as monoterpene and sesquiterpene hydrocarbons, oxygenated monoterpenes and sesquiterpenes, esters, aldehydes, ketones, alcohols, phenols, and oxides [2,3,4]. The chemical composition of EOs can vary from plant to plant and even in the same species and depends on several factors such as post-harvest conservation conditions [5], extraction methods [6,7,8] and times [9,10], microclimate, and site in which the plant is growing [11,12]. EOs contribute to the plant relations with environment and with other organisms, and humans and animals take advantage of the abundance of such bioactive molecules from the plant kingdom [4]. Numerous papers deal with the biological activities of the EOs such as antioxidant and anti-inflammatory properties, antibacterial and antifungal activities, immunomodulatory effects, and cytotoxic activities against different cancer cell lines [13,14,15,16,17,18,19]. Gymnosperms, the Cupressaceae, and the Pinaceae families produce economically important EOs [20]. The Pinaceae family is the largest family of non-flowering seed plants and comprises 11 genera and approximately 230 species of trees, rarely shrubs, which are widely distributed in the Northern Hemisphere [21,22]. The biological activities of Pinaceae EOs reflect the richness in their chemical composition. Antioxidant, antibacterial, antifungal, insect larvicidal, anti-inflammatory, and antiproliferative activities are reported for different genus of the Pinaceae family [16,23,24,25,26,27,28,29,30].

In our searching and studying of natural compounds, in the present paper, we investigated and compared the chemical composition and the antimicrobial and antioxidant properties of the vapor and liquid phase of four Pinaceae EOs from *Pinus cembra* L. and *Pinus mugo* Turra, which belong to the Pinus genus, and *Picea abies* L. and *Abies alba* M., which belong to the Picea and Abies genus, respectively.

## 2. Results

### 2.1. Liquid and Vapor Phases EOs Chemical Composition

By Gas Chromatography-Mass Spectrometry (GC/MS) and Headspace (HS)-GC/MS analysis, the composition of the vapor and liquid phase of all EOs was described. Twenty components were identified in *P. cembra* and *P. mugo* EOs, and they are listed in Table 1. The most abundant component was α-pinene (44.0% when using GC/MS and 65.6% when using HS/GC-MS) followed by γ-terpinene (19.7% GC/MS; 11.0% HS/GC-MS), limonene (14.8% GC/MS and 8.2%; HS/GC-MS) and β-pinene (12.5% GC/MS; 12.4% HS/GC-MS) in *P. cembra* EO. On the contrary, β-pinene (43.3% GC/MS; 42.3% HS/GC-MS) was the major compound in *P. mugo*. EO followed by α-pinene (16.6% GC/MS; 31.6% HS/GC-MS) and limonene (9.5% GC/MS; 7.8% HS/GC-MS). β-Phellandrene (16.0%) as well as other minor compounds such as p-cymenene (0.1%), copaene (0.1%), and bornyl acetate (3.0%) appeared only in the liquid phase of *P. mugo* EO. On the other hand, α-phellandrene (0.7%) was detected only in *P. mugo* vapor phase EO. In particular, in the vapor phase of both EOs, the components from N° 11 to N° 20 were missing except for β-caryophyllene (0.1%), which was detected in *P. mugo* EO.

Twenty-one components were identified in *P. abies* and *A. alba* EOs, and they are listed in Table 2. β-Pinene was the principal compound in *P. abies* EO (20.2% when using GC/MS and 34.5% when using HS/GC-MS), while α-pinene (30.8% GC/MS; 51.3% HS/GC-MS) was the principal compound in *P. abies* EO. The second most abundant component was α-pinene (20.2% and 34.5%) in *P. abies* EO and limonene (32.5% and 19.0%) in *A. alba* EO when using GC/MS and HS/GC-MS, respectively. p-Cymene (0.2%; 0.1%), camphor (1.2%; 0.2%), and borneol (2.1%; 0.2%) were detected only in the liquid and vapor phase, respectively of *P. abies* EOs. α-Himachalene (0.3%), citronellol acetate (0.4%), humulene (1.6%), and caryophyllene oxide (0.1) appeared only in the liquid phase of *A. alba* EO. Lastly, in the vapor phase of both EOs, the components from N° 12 to N° 21 were missing except for borneol (0.2%), which was detected in *P. abies* EO.

**Figure 1:**
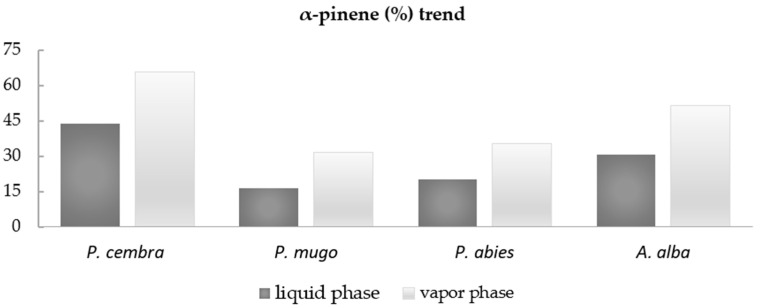
Bar graph of α-pinene percentage trend in liquid and vapor phase EOs.

Among the most abundant compounds, particular attention was paid to α-pinene, as it always reached higher percentages in the vapor phase than in the liquid phase of the investigated EOs. The compared values are as follows: (44.0% vs. 65.6%), (16.6% vs. 31.6%), (20.2% vs. 35.5%), and (30.8% vs. 51.3%) liquid and vapor phase in *P. cembra*, *P. mugo*, *P. abies*, and *A. alba* EOs, respectively (Figure 1).

### 2.2. Antibacterial Activities of P. Cembra, P. Mughus, P. Abies, and A. Alba EOs

The antibacterial activities of the Pinaceae EOs were evaluated for three Gram-negative (*Escherichia coli*, *Pseudomonas fluorescens*, and *Acinetobacter bohemicus*) and two Gram-positive bacteria (*Kocuria marina* and *Bacillus cereus* using micro dilution assay to determine Minimum Inhibitory Concentration (MIC) and the Minimal Bactericidal Concentration (MBC), and the MBC/MIC ratio defines an agent as bacteriostatic when the MBC/MIC ratio > 4 and as bactericidal when the MBC/MIC ratio ≤ 4 [31]. Furthermore, the disc diffusion assay by contact with the essential oil determined the diameter of bacterial growth inhibition zone (IZ), and the vapor phase test determined the antibacterial growth inhibition zone (Vapor IZ) by more volatile molecules of the EO in a preservative atmosphere. The antibacterial results of the tested EOs are summarized in Table 3, Table 4, Table 5 and Table 6 reporting the MIC, MBC, MBC/MIC ratio, IZ, and vapor IZ following the treatments for each bacterial strain. In Table 3, the treatment with *P. cembra* EO showed MIC and MBC values of 53.12 mg/mL for *E. coli*, *P. fluorescens*, and *K. marina*, while MIC values were 26.56 mg/mL for *A. bohemicus* and *B. cereus*, and MBC values were 26.56 mg/mL and 53.12 mg/mL, respectively. MBC/MIC ratio defined the *P. cembra* EO as bactericidal against all bacterial strains. No effects were observed with the disc diffusion assay and with the vapor phase test for *P. cembra* EO against *E. coli* and *P. fluorescens*. The IZ and vapor IZ values were 17.67 ± 0.58 mm and 67.33 ± 2.52 mm for *A. bohemicus*, 9.33 ± 0.58 mm and 80.00 ± 0.00 mm for *K. marina*, and 11.67 ± 1.15 mm and 80.00 ± 0.00 mm for *B. cereus*, respectively.

Table 4 summarizes the antibacterial tests for *P. mugo* EO. MIC and MBC values were 52.16 mg/mL for *E. coli*, *P. fluorescens*, and *K. marina*, while MIC and MBC values were 26.08 mg/mL for *A. bohemicus* and MIC and MBC values were 26.08 mg/mL and 52.16 mg/mL for *B. cereus*, respectively. The MBC/MIC ratio defined as bactericidal the *P. mugo* EO against all bacterial strains. No effects were observed by the disc diffusion assay and by the vapor phase test for *P. cembra* EO on *P. fluorescens*. *P. mugo* EO was not highly active against *E. coli* with an IZ value of 9.67 ± 0.58 mm, while no growth inhibition zone was observed by the vapor phase test. Higher antibacterial activity was observed for the other bacterial strains: IZ and vapor IZ values were 25.33 ± 4.51 mm and 41.00 ± 10.15 mm for *A. bohemicus*, 11.33 ± 1.15 mm and 80.00 ± 0.00 mm for *K. marina*, and 15.67 ± 1.15 mm and 76.67 ± 5.77 mm for *B. cereus*, respectively. The *P. mugo* EO vapor phase was more active than the liquid phase against *A. bohemicus*, *K. marina*, and *B. cereus.*

*P. abies* EO antibacterial activity is reported in Table 5. MIC and MBC values were 53.12 mg/mL for *E. coli*, *P. fluorescens,* and *K. marina*. For *A. bohemicus*, a lower MIC value was observed (13.28 mg/mL), whereas MBC was 26.56 mg/mL. The antibacterial activity for *B. cereus* was 26.56 mg/mL and 53.12 mg/mL for the MIC and MBC values, respectively. As obtained by the MBC/MIC ratio, *P. abies* EO was bactericidal against all bacterial strains. No effects were observed by the disc diffusion assay and by the vapor phase test for *P. abies* EO on *P. fluorescens* and *E. coli*. IZ and vapor IZ values were 18.67 ± 1.53 mm and 76.67 ± 5.77 mm for *A. bohemicus*, 9.67 ± 1.15 mm and 80.00 ± 0.00 mm for *K. marina* and 11.67 ± 1.53 mm and 80.00 ± 0.00 mm for *B. cereus*, respectively. The *P. abies* EO vapor phase was more active rather than the liquid phase against *A. bohemicus*, *K. marina*, and *B. cereus*.

The results for *A. alba* antibacterial activity are reported in Table 6. MIC and MBC values were 51.28 mg/mL for *E. coli*, *P. fluorescens*, and *K. marina*. Lower MIC and MBC values were found for *A. bohemicus* and for *B. cereus* (12.82 mg/mL and 25.64 mg/mL, respectively). The MBC/MIC ratio defined as bactericidal the *A. alba* EO against all bacterial strains. No effects were observed in the disc diffusion assay and in the vapor phase test for *A. alba* EO on *E. coli* and *P. fluorescens*. Higher antibacterial activity was detected for the other bacterial strains: IZ and vapor IZ values were 19.67 ± 0.58 mm and 80.00 ± 00 mm for *A. bohemicus*, 7.67 ± 1.15 and 80.00 ± 0.00 for *K. marina*, and 15.00 ± 2.65 and 66.67 ± 11.55 for *B. cereus*, respectively. The vapor phase test revealed that the activities of the *A. alba* EO against *A. bohemicus*, *K. marina,* and *B. cereus* were higher than those of the liquid phase. 

### 2.3. Antioxidant Activity

To determine the antioxidant activity of the four Pinaceae EOs, 2,2-Diphenyl-1-picrylhydrazyl (DPPH) scavenging activity and 2,2′-azinobis (3- ethylbenzothiazoline-6-sulfonic acid) diammonium salt (ABTS) radical scavenging assay, based on the reaction of the potential antioxidant with colored radicals, were carried out. The antioxidant activity results are reported in Table 7. In all EOs, a concentration-dependent antioxidant capacity was found. In both tests, *P. mugo* EO showed the highest antioxidant activity than the other Pinaceae EOs. This EO exhibits lower IC_50_ values (3.08 µg/mL and 43.08 µg/mL for DPPH and ABTS assays, respectively) and higher TEAC values (7.65 mol/mg and 14.01 mol/mg for DPPH and ABTS assays, respectively). The second effective essential oil was the *A. alba* EO with IC_50_ values of 7.84 µg/mL and 44.23 µg/mL and TEAC values of 1.63 mol/mg and 13.26 mol/mg in the DPPH method and ABTS method, respectively. The TEAC values of *P. cembra* and *P. abies* EOs were almost identical: 1.63 mol/mg and 1.68 mol/mg in the DPPH assay, respectively and 13.26 mol/mg for both the EOs in the ABTS assay. Taking into account the ABTS test, the IC_50_ amount was 44.90 µg/mL in *P. cembra* EO and 45.00 µg/mL in *A. alba* EO. In the DPPH test, both EOs showed similar IC_50_ values, too (13.01 µg/mL for *P. cembra* and 13.05 µg/mL for *P. abies*). *A. alba* EO remains the least effective in antioxidant capacity of the analyzed Pinaceae EOs. 

## 3. Discussion

The chemical profile of both the vapor and liquid phase and the antibacterial and antioxidant activities of four Pinaceae EOs, obtained from needles by steam distillation, were investigated using different kinds of techniques and assays. In the literature, a few papers reporting the Pinaceae EOs chemical composition are present, and no report describes the volatile composition of the vapor phase of the conifer-derived EOs by HS-GC/MS, as we applied for our investigation. In our investigations, the chemical constituents resulted primarily monoterpenoids and their contents were higher in the vapor phases of *P. cembra* (99.9%) and *P. mugo* (100.0%) EOs than the vapor phases of the *P. abies* (97.9%) and *A. alba* (95.8%) EOs. The major compounds of the *P. cembra* EO were α-pinene (44.0%), γ-terpinene (19.7%), limonene (14.8%), and β-pinene (12.5%). Similar composition was described by Lis et al. [32], where the needle oil was dominated by α-pinene (48.4%), limonene (7.5%), and β-phellanderene (3.1%); α-pinene was also the major component (69.14%) in needle EO of *P. cembra* growing in Romania [33]. The composition of the EO from twig tips with needles of the *P. cembra* L. growing in Salzburg Alps was represented by α-pinene (43.9–48.3%), β-phellandrene (13.1–17.2%), and β-pinene (6.6–9.3%) [34]. *Pinus cembra* needles EO from Slovakia consisted of α-pinene (53.2%), limonene (11.4%), and β-phellandrene (9.4%) [35].

The main components of *P. mugo* EO were β-pinene (43.3%), α-pinene (16.6%), β-phellandrene (16.0%), and limonene (9.5%) with a low percentage of β-caryophyllene (3.6%). A different composition was reported for *P. mugo* EO from needles growing in Poland where 3-carene (23.8 %), myrcene (22.3 %), and α-pinene (10.3 %) resulted as the main components [36]. 3-Carene (31.73%) was also the major compound in EO of *P. mugo* from Kosovo [37], followed by α-pinene (19.95%) and β-phellandrene (13.49%). *P.mugo* needles EOs from Macedonian [38] and Serbia [39] mainly consisted of Δ^3^-carene (amount up to 35% and 23.9%), α- and β-pinene (up to 20% and 17.9%) and β-phellandrene (amount about 15% and 7.2%), respectively.

In *P. abies* EO, we found β-pinene (44.7%) as the most abundant component followed by α-pinene (20.2%), limonene (14.2%), and camphene (7.2%). A different composition has been described for the EOs from shoots of *P. abies* that grow wild in different locations of Romania, which are characterized by limonene (from 6.27% up to 12.98%), camphene (from 3.89% up to 14.07%), α-pinene (from 2.44% up to 10.42%), and β-myrcene (from 0.44% up to 10.12%) [40].

The chemical composition of *A. alba* EO showed two components such as limonene and α-pinene with a similar percentage (32.5% and 30.8%) followed by camphene (11.2%) and β-pinene (7.5%). The same compounds were listed with an inverted trend in *A. alba* EO from Montenegro where β-pinene (32.8%) was the major component followed by α-pinene (17.3%) and camphene (16.7%) [41]. On the contrary, α-limonene (about 70%) and α-pinene (57%) were the major compounds in *A. alba* EO from seeds and cones respectively [42]. In *A. alba* EO from Poland, limonene was the component with the higher percentage (82.9%) detected in seed EO, whereas α-pinene (50.0%) was the main component in cone EO [43]. According to the literature [44] and on the basis of the reported data, it becomes evident that the chemical composition of the EOs from species belonging to the Pinaceae family can depend by multiple factors such as part of the plant examined, its geographic origin, and also, extraction methods and storage [45].

MIC and MBC values defined by the microwell dilution method were tested against *E. coli*, *P. fluorescens* and *A. bohemicus* Gram-negative bacteria strains and *K. marina* and *B. cereus* Gram-positive bacteria strains. The lowest MIC (13.28 mg/mL) and MBC (26.56 mg/mL) values, which correspond to the highest antibacterial activities, were reported for *P. abies* EO against *A. bohemicus* and for *A. alba* EO against *A. bohemicus* and *B. cereus* with 12.82 mg/mL (MIC) and 25.64 mg/mL (MBC).

The increase of antibiotically resistance phenomenon in human and animal pathologies has determined the intensification of research on new natural antimicrobial substances [19,46,47], and in this view, several studies were carried out to investigate the biological activities of Pinaceae EOs and the roles of their molecules. *P. abies* EO extracted by supercritical carbon dioxide was investigated for antimicrobial properties on *E. coli* using the isothermal calorimetry technique, and it inhibited the growth and interfered with the metabolic activity of the microorganism [48]. Kartnig et al. [49] determined the antibacterial activities of the essential oils of young pine shoots on different bacterial strains also from human patients, and significant activities were revealed against G+ bacteria strains and *Candida* species tested. Apetrei et al. [25] reported that needles and twigs essential oils of *Pinus cembra* showed high activity against *Sarcina lutea* and *Staphylococcus aureus* and no activity against *B. cereus*, *E. coli* and *Pseudomonas aeruginosa*.

The antibacterial activities of the four Pinaceae EOs were also confirmed by agar diffusion and disk volatilization methods by which the IZ and vapor IZ were measured in mm of inhibition halos. For all the tested EOs, the vapor phases were more active than the liquid phases, showing the inhibition halos from 41.00 ± 10.15 mm to 80.00 ± 0.00 mm for three bacterial strains (*A. bohemicus*, *K. marina*, and *B. cereus*). Concerning *E. coli* and *P. fluorescens*, a very low or null activity was reported. The results showed high activities of the EOs against *A. bohemicus*, *K. marina*, and *B. cereus* and a scarce or null activity against *E. coli* and *P. fluorescens*. The highest activities obtained by vapor phases of all EOs against *A. bohemicus*, *K. marina*, and *B. cereus* could be related with the presence of α-pinene. In the graph bar (Figure 1), the relative percentages of α-pinene were reported. It reached higher percentages in the vapor phase than in the liquid phase of all investigated EOs. In particular, liquid and vapor phase values were as follows: (44.0% vs. 65.6%), (16.6% vs. 31.6%), (20.2% vs. 35.5%), and (30.8% vs. 51.3%), in *P. cembra*, *P. mugo*, *P. abies*, and *A. alba* EOs respectively. These results suggest that α-pinene could play an important role for the detected antibacterial activity. Some papers reported α-pinene from Pinaceae EOs as the main compound showing good biological activity; it was the principal constituent (5.2–37.0%) in five Moroccan Pinus species EOs [50] and in *Pinus peuce* Griseb. EOs (12.89–27.34%) growing on three different locations in R. Macedonia [51].

Different studies confirmed the antibacterial properties of α-pinene [52]. Freitas et al. [53] reported that α-pinene has antibacterial and antibiotic-modulating activities against *S. aureus;* it also increases the activity of norfloxacin against *E. coli* and norfloxacin and gentamicin against *S. aureus*. Furthermore, Hippeli et al. [54] described an anti-inflammatory potential of *P. mugo* EOs and its main compound α-pinene, while Cole et al. [55] showed anti-proliferative activity on the MCF-7 cell line. On the other hand, Kurti et al. [37] attributed the antimicrobial activities of some Pinus species EOs from Kosovo to the hexane/diethyl ether fractions, which were mainly composed by oxygenated monoterpenes.

In the present study, the susceptibility of bacteria does not seem to be related with the features of the cell Gram-positive and Gram-negative bacteria wall structure, since the more sensitive bacteria strains, *A. bohemicus*, *K. marina*, and *B. cereus* do not belong to the same group. Generally, Gram-negative are more resistant than Gram-positive bacteria, because the cell wall does not allow the entrance into the cell of hydrophobic molecules present in the essential oils [56,57], although some exceptions have been shown [58,59]. In a comparative study of the essential oils from four Pinus species [30], it was found that the sensitivity of the tested bacterial pathogens cannot be related with the cell wall structure. Different mechanisms of action can explain the EOs antimicrobial activities, and their wide variety of molecular components can act at multiple levels [60].

The DPPH and ABTS assays demonstrated a significant antioxidant activity for all Pinaceae EOs. *P. mugo* EO was the more active with an IC_50_ 3.08 ± 0.65 and 43.08 ± 6.95 µg/mL for DPPH and ABTS assays, respectively. The values expressed in Trolox equivalent (TEAC) confirmed identical results. A comparative investigation has been carried out on *P. halepensis* EO chemical composition and antioxidant activities, with respect to the impact of geographic variation and environmental conditions [61].

The variety of compounds that are present in the investigated EOs confers them numerous biological properties, and their antioxidant activities could be related to the presence of monoterpenes. Wang et al. [62] studied the antioxidant activities of seven terpenoids found in wine, and among the tested compounds, α-pinene and limonene had the highest DPPH free radical scavenging and the highest reducing power. Wojtunik-Kulesza [63] reviewed the monoterpenes biological properties and antioxidant activities of α-pinene were also reported.

## 4. Materials and Methods

### 4.1. Materials

*P. cembra* L., *P. mugo* Turra, *A. alba* M., and *P. abies* L. bio essential oils (IT BIO 013 n° BZ-43509-AB) from needles growing in Alto Adige, Italy were obtained by steam distillation for 6 h extraction time and were directly provided by Bergila GmbH Srl (Falzes/Issengo-Bolzano). Methanol, 2,2-Diphenyl-1-picrylhydrazyl (DPPH), 6-hydroxy-2,5,7,8-tetramethylchroman-2-carboxylic acid (Trolox), 2,2′-azinobis (3-ethylbenzothiazoline-6-sulfonic acid) diammonium salt (ABTS), potassium persulfate (K_2_S_2_O_8_), LB Broth with Agar and Thiazolyl Blue Tetrazolium Bromide (MTT) were from Sigma-Aldrich (Darmstadt, Germany). Gentamicin sulfate was purchased from Biochrom PAN-Bio-Tech GmbH (Aidenbach, Germany).

### 4.2. Gas Chromatography–Mass Spectrometry (GC–MS) Analysis

To describe the chemical composition of the EOs, a gas chromatograph with a flame ionization detector (FID) directly coupled to a mass spectrometer (MS) Perkin Elmer Clarus 500 model (Waltham, MA, USA) was used. The GC was equipped with a Restek Stabilwax (fused-silica) polar capillary column. Helium was used as carrier gas at a flow rate of 1 mL/min. The injector was set to a 280 °C, and the oven temperature program was as follows: isothermal at 60 °C for 5 min, then ramped to 220 °C at a rate of 6 °C min^−1^, and finally isothermal at 220 °C for 20 min. One uL of EO was diluted in 1 mL of methanol, and the injection volume was 1 μL. The Electron Impact-Mass Spectrometer (EI-MS) mass spectra were recorded at 70 eV (EI) and were scanned in the range of 40–500 *m*/*z*. The ion source and the connection parts temperature was 220 °C. The injector split ratio was 1:20. The GC-TIC mass spectra were obtained by the TurboMass data analysis software (Perkin Elmer). The identification of components was performed by matching their mass spectra with those stored in the Wiley and NIST 02 mass spectra libraries database. Furthermore, the linear retention indices (LRIs) (relative to C8–C30 aliphatic hydrocarbons, injected in the column at the same operating conditions described above) were calculated and compared with available retention data present in the literature. The relative percentages of all identified components were obtained by peak area normalization from GC-FID chromatograms without the use of an internal standard or correction factors and expressed in percentages. All analyses were repeated twice.

### 4.3. Headspace GC-MS Analysis

The volatile chemical profile of essential oils was carried out with a Perkin Elmer Headspace Turbomatrix 40 (Waltham, MA, USA) autosampler connected to GC-MS [64,65]. One mL of the each EO was placed in 20 mL vials sealed with headspace PTFE-coated silicone rubber septa and caps. To optimize the headspace procedure for the determination of volatile organic compounds (VOCs), more operative parameters were optimized. The gas phase of the sealed vials was equilibrated for 20 min at 60 °C and was followed immediately by compound desorption into GC injector in splitless mode. Quantification of compounds was performed by GC-FID in the same conditions described in the previous paragraph.

### 4.4. Antibacterial Activities of the Pinaceae Essential Oils

The antibacterial activities were investigated by using different methods, the Minimal Inhibitory Concentration (MIC), the Minimal Bactericidal Concentration (MBC), the agar diffusion method, and Vapor Phase Test (VPT).

#### 4.4.1. Bacterial Strains

Five bacterial strains from the culture collections of the Plant Cytology and Biotechnology Laboratory of Tuscia University were tested to evaluate the antibacterial activities of *P. cembra* L., *P. mugo* Turra, *A. alba* M., and *P. abies* L. essential oils: *Escherichia coli* ATCC 25922, *Pseudomonas fluorescens* ATCC 13525, and *Acinetobacter bohemicus* DSM 102855 among Gram-negative and *Kocuria marina* DSM 16420 and *Bacillus cereus* ATCC 10876 among Gram-positive. All tested bacterial strains were maintained on LB broth (10 g tryptone, 5 g yeast extract, 10 g NaCl per liter, autoclaved at 121 °C for 20 min) with agar. Bacteria cultures were maintained at two different temperatures: 26 °C for *P. fluorescens*, *A. bohemicus,* and *B. cereus* and 37 °C for *K. marina* and *E. coli*. All inocula were prepared with fresh cultures plated the day before the test.

#### 4.4.2. Minimum Inhibitory Concentration (MIC)

The MIC is defined as the lowest concentration of antimicrobial agent that completely inhibits the growth of the microorganism as detected by the unaided eye and was carried out according to the microwell dilution method. Briefly, 12 dilutions of the four essential oils in LB broth (for *P. cembra* from 53.12 to 0.01 mg/mL; for *P. mugo* from 52.16 to 0.01 mg/mL; for *A. alba* form 51.28 to 0.01 mg/mL and for *P. abies* from 53.12 to 0.01 mg/mL), a control with the same percentage of DMSO (from 6.25% to 0.003%) in Lysogeny broth, a growth control without treatments, a positive control with gentamicin diluted from 100 to 0.05 µg/mL, and a sterility control without bacteria were plated on 96 microwell plates. Then, 50 µL of bacterial inoculum, 10^6^ CFU/mL, were added in each well, except for the sterility control, and the plates were incubated for 24 h at the corresponding temperature. The visualization of the inhibition activity was obtained by 20 µL of a solution of 3-(4,5-dimethylthiazol-2-yl)-2,5-diphenyltetrazolium bromide (200 µg/mL, MTT) added to each well. The assay was carried out in triplicate. The MBC/MIC ratio was reported to interpret the activity of the essential oil, and an antimicrobial agent is considered bacteriostatic when the ratio MBC/MIC > 4 and bactericidal when the ratio MBC/MIC is ≤4 [31].

#### 4.4.3. Minimum Bactericidal Concentration (MBC)

To verify the lowest concentration at which the tested essential oils kill the bacterial cells, which is defined the Minimum Bactericidal Concentration (MBC), 10 µL of the last four dilutions from microwell dilution method in which no bacteria growth was observed were plated on a Petri plate with LB agar and incubated for 24 h. The concentration at which no growth on agar was observed defined MBC values. The assay was carried out in triplicate.

#### 4.4.4. Agar Diffusion Method

To determine the diameter of the halo inhibition of the bacteria growth induced by *P. abies*, *A. alba*, *P. cembra*, and *P. mugo* essential oils, the bacterial strains were suspended in LB broth to obtain a turbidity of 0.5 McFarland (approximately 10^8^ Colony-Forming Unit/mL—CFU/mL) and then plated on LB broth with agar in a Petri plate. Sterile disks (6 mm diameter, Oxoid) were placed on the agar and impregnated with 10 µL of samples. Two µL of gentamicin from a stock solution (10 mg/mL) was used as a positive control After 24 h, the inhibitory activities of each essential oil were recorded as mm of halo diameter without growth [58] using a vernier caliper rule. The mean and the respective standard deviation (SD) of the measured halo in three independent experiments were recorded.

#### 4.4.5. Vapor Phase Test (VPT)

The antibacterial activity of the Pinaceae essential oils in the vapor phase was evaluated by the modified disk volatilization method [66,67]. LB agar were poured into an 80 mm plastic Petri dish and a lower amount into its cover. Each bacterial suspension containing 10^8^ CFU/mL was plated on the LB agar medium. Then, 10 µL of tested essential oils were added to a 6 mm sterile disk and placed on agar in the covered Petri plate. Liquid LB agar was put in the space between the cover and the base of the Petri dishes to facilitate the sealing and to prevent any vapor leakage. The Petri plates were incubated for 24 h in an inverted position, and afterwards, the inhibition halos were measured. Negative controls were carried out without the essential. All VPTs were carried out in triplicate.

### 4.5. Antioxidant Activity

To assess the antioxidant activity of the four Pinaceae essential oils, DPPH radical scavenging activity and ABTS radical scavenging assay, which are based on the reaction of the potential antioxidant with colored radicals, were carried out.

#### 4.5.1. DPPH Scavenging Activity Assay

In DPPH radical scavenging assay, the Pinaceae essential oils antioxidant activities were calculated against the 1,1-diphenyl-2-picrilidrazil radical (DPPH•) using the method described by Sanchez-Moreno et al. [68]. First, 100 µL of fresh solution of a solid crystalline DPPH• (0.2 mM) in methanol were added to 100 µL of 12 geometric dilutions in methanol of each essential oil inside a 96-well plate. Geometric dilutions of the samples in methanol were used as sample blanks. In blank DPPH samples, essential oils were omitted. As a positive control, dilutions were prepared starting from Trolox solution (1 mM) in methanol. The samples were incubated for 30 min in the dark at room temperature, and the absorbances decreases were measured at 517 nm using a Tecan Sunrise^TM^ UV-vis spectrophotometer. The assay was repeated three times.

#### 4.5.2. ABTS Radical Scavenging Assay

The radical scavenging activities of the Pinaceae essential oils were also calculated using the ABTS (2,2′-azino-bis (3-ethylbenzothiazoline-6-sulfonic acid) diammonium salt) assay described by Re et al. [69] with some modifications. The radical cation ABTS+• was produced by reacting ABTS aqueous solution (7 mM) with K_2_S_2_O_8_ (140 mM) following an incubation for 16 h in the dark at room temperature before use. The ABTS+• solution was diluted with ethanol to reach an absorbance of 0.70 ± 0.02 at 734 nm, and 1980 µL was mixed with 20 µL of the essential oil dilutions in ethanol. The resulting solutions were incubated for 5 min at room temperature. Afterwards, the absorbances were measured at 734 nm using a Jasco V-630 UV-Visible spectrophotometer and using Spectra Manager^TM^ software. Furthermore, the absorbance of the ABTS+• blank, consisting of 20 µL of ethanol dissolved in 1980 µL of ABTS+• solution, was measured. The assay was repeated three times.

#### 4.5.3. IC_50_ and TEAC Calculation

Trolox and samples calibration curves were obtained by plotting the inhibition ratio against sample concentrations. The inhibition ratio was calculated using the following formula:

(1) $$IR\%\;=\;\frac{A\;blank-A\;sample}{A\;blank}\;\times \;100.$$

The IC50 parameter was calculated using the sample calibration curve. A lower value of the IC50 parameter correspond to a lower concentration of the EO that can scavenge 50% of DPPH· molecules; therefore, it indicates a higher antioxidant activity.

The Trolox equivalent antioxidant capacity (TEAC) index was obtained from the ratio between the Trolox IC50 (µM) and the sample IC50 (mg/L):

(2) $$TEAC\;=\;\frac{{IC}_{50_{trolox}}}{{IC}_{50_{sample}}}.$$

### 4.6. Statistical Analysis

The results were expressed as means ± standard deviation (SD). The one-way analysis of variance test (ANOVA) using GraphPad Prism software (GraphPad Prism 5.0, GraphPad Software, Inc., San Diego, CA, USA) was used to evaluate statistical discrepancies between the groups (*p* values < 0.05).

## 5. Conclusions

In this study, for the first time, the chemical composition of the liquid and vapor phase of four Pinaceae EOs was investigated by the HS-GC/MS technique. The results of analyses showed that these EOs are rich in monoterpenoids and highlight that α-pinene, one of the main compounds, is more abundant in the vapor phase of each oil than in the liquid phase. The antimicrobial and antioxidant activities were also reported and compared. The vapor phase of each EO resulted more active against the investigated bacterial strains.

The biological effects of the Pinaceae EOs combined with their bioavailability makes them promising sources for possible application in different fields such as pharmacology, pharmacognosy, and phytochemistry.

## Figures and Tables

**Table 1:** Chemical composition (%) of liquid and vapor phases of *P. cembra* and *P. mugo* EOs.

N°	COMPONENT ^1^	LRI ^2^	LRI ^3^	*Pc*(%) ^4^	*Pc*(%) ^5^	*Pm*(%) ^6^	*Pm*(%) ^7^
1	α-pinene	1019	1021	44.0	65.6	16.6	31.6
2	camphene	1062	1065	1.6	2.3	1.6	2.9
3	β-pinene	1098	1099	12.5	12.4	43.3	42.3
4	β-thujene	1120	1118	0.3	0.2	0.3	-
5	α-phellandrene	1158	1160	-	-	-	0.7
6	limonene	1197	1198	14.8	8.2	9.5	7.8
7	β-phellandrene	1210	1207	-	-	16.0	-
8	γ-terpinene	1270	1241	19.7	11.0	0.3	13.3
9	p-cymene	1270	1268	0.1	Tr	0.2	0.2
10	terpinolene	1285	1282	0.5	0.2	2.1	1.1
11	p-cymenene	1431	1435	-	-	0.1	-
12	copaene	1491	1487	-	-	0.1	-
13	bornyl acetate	1571	1567	-	-	3.0	-
14	thymol methyl ether	1576	1575	1.2	tr	-	-
15	β-caryophyllene	1620	1619	0.4	-	3.6	0.1
16	α-terpineol	1655	1655	0.2	-	0.2	tr
17	humulene	1670	1667	0.5	-	1.1	-
18	γ-muurolene	1674	1673	0.4	-	0.3	-
19	α-muurolene	1691	1690	2.4	-	0.9	-
20	δ-cadinene	1760	1758	1.4	-	0.5	-
	SUM			100.0	99.9	99.7	100.0
	Monoterpenes			93.7	99.9	93.2	100.0
	Sesquiterpenes			5.1	-	6.5	-
	Other			1.2	-	-	-

^1^ The components are reported according their elution order on a polar column; ^2^ Linear retention indices measured on polar column; ^3^ Linear retention indices from the literature; ^4^ Percentage values of *P. cembra* EO components (%); ^5^ Percentage values of *P. cembra* EO components (vapor phase); ^6^ Percentage mean values of *P. mugo* EO components (%); ^7^ Percentage mean values of *P. mugo* EO components (vapor phase); -Not detected; tr: traces (mean value < 0.1%).

**Table 2:** Chemical composition (%) of liquid and vapor phases of *P. abies* and *A. alba* essential oils (EOs).

N°	COMPONENT ^1^	LRI ^2^	LRI ^3^	*Pa*(%) ^4^	*Pa*(%) ^5^	*Aa*(%) ^6^	*Aa*(%) ^7^
1	santene	980	984	0.7	1.9	1.4	4.2
2	α-pinene	1019	1021	20.2	34.5	30.8	51.3
3	camphene	1062	1065	7.2	10.5	11.2	16.5
4	β-pinene	1098	1099	44.7	43.8	7.5	7.3
5	limonene	1197	1198	14.2	8.0	32.5	19.0
6	γ-terpinene	1240	1241	0.3	0.1	1.1	1.2
7	p-cymene	1270	1268	0.2	0.1	-	-
8	terpinolene	1285	1282	0.6	0.2	0.5	0.1
9	α-longipinene	1480	1477	0.3	-	0.9	-
10	camphor	1506	1507	1.2	0.2	-	-
11	bornyl acetate	1571	1567	3.7	0.3	4.2	0.4
12	longifolene	1585	1583	1.1	-	0.6	-
13	β-caryophyllene	1620	1619	1.3	-	5.8	-
14	α-himachalene	1641	1637	-	-	0.3	-
15	citronellol acetate	1646	1644	-	-	0.4	-
16	α-terpineol	1655	1655	0.4	tr	0.2	-
17	humulene	1670	1667	-	-	1.6	-
18	γ-muurolene	1674	1673	0.4	-	0.3	-
19	borneol	1677	1675	2.1	0.2	-	-
20	δ-cadinene	1760	1758	0.9	-	0.6	-
21	caryophyllene oxide	1895	1892	-	-	0.1	-
	SUM			99.5	99.8	100.0	100.0
	Monoterpenes			94.8	97.9	88.4	95.8
	Sesquiterpenes			4.0	0.2	9.9	-
	Other			0.7	1.9	1.7	4.2

^1^ The components are reported according their elution order on polar column; ^2^ Linear retention indices measured on polar column; ^3^ Linear retention indices from the literature; ^4^ Percentage mean values of *P. abies* EO components (%); ^5^ Percentage mean values of *P. abies* EO components (vapor phase); ^6^ Percentage mean values of *A. alba* EO components (%); ^7^ Percentage mean values of *A. alba* EO components (vapor phase); -Not detected; tr: traces (mean value < 0.1%).

**Table 3:** Antibacterial activity of *P. cembra* EO.

Strains	*Pinus cembra*				
	MIC ^1^	MBC ^2^	MBC/MIC Ratio	IZ ^3^	Vapor IZ ^4^
*E. coli*	53.12	53.12	1	0.00	0.00
*P. fluorescens*	53.12	53.12	1	0.00	0.00
*A. bohemicus*	26.56	26.56	1	17.67 ± 0.58	67.33 ± 2.52
*K. marina*	53.12	53.12	1	9.33 ± 0.58	80.00 ± 0.00
*B. cereus*	26.56	53.12	2	11.67 ± 1.15	80.00 ± 0.00

^1^ Minimal Inhibitory Concentration expressed in mg/mL of EO treatment; ^2^ Minimal Bactericidal Concentration expressed in mg/mL of EO treatment; ^3^ Growth inhibition zone by disc diffusion assay expressed in mm; ^4^ Growth inhibition zone by vapor phase test expressed in mm. Values are expressed as means ± SD. *p* < 0.05 from one-way analysis of variance test (ANOVA).

**Table 4:** Antibacterial activity of *P. mugo* EO.

Strains	*Pinus mugo*				
	MIC ^1^	MBC ^2^	MBC/MIC Ratio	IZ ^3^	Vapor IZ ^4^
*E. coli*	52.16	52.16	1	9.67 ± 0.58	0.00
*P. fluorescens*	52.16	52.16	1	0.00	0.00
*A. bohemicus*	26.56	26.56	1	25.33 ± 4.51	41 ± 10.15
*K. marina*	52.16	52.16	1	11.33 ± 1.15	80.00 ± 0.00
*B. cereus*	26.56	52.16	2	15.67 ± 1.15	76.67 ± 5.77

^1^ Minimal Inhibitory Concentration expressed in mg/mL of EO treatment; ^2^ Minimal Bactericidal Concentration expressed in mg/mL of EO treatment; ^3^ Growth inhibition zone by disc diffusion assay expressed in mm; ^4^ Growth inhibition zone by vapor phase test expressed in mm. Values are expressed as means ± SD. *p* < 0.05 from one-way analysis of variance test (ANOVA).

**Table 5:** Antibacterial activity of *P. abies* EO.

Strains/Origin	*Picea abies*				
	MIC ^1^	MBC ^2^	MBC/MIC Ratio	IZ ^3^	Vapor IZ ^4^
*E. coli*	53.12	53.12	1	0.00	0.00
*P. fluorescens*	53.12	53.12	1	0.00	0.00
*A. bohemicus*	13.28	26.56	2	18.67 ± 1.53	76.67 ± 5.77
*K. marina*	53.12	53.12	1	9.67 ± 1.15	80.00 ± 0.00
*B. cereus*	26.56	53.12	2	11.67 ± 1.53	80.00 ± 0.00

^1^ Minimal Inhibitory Concentration expressed in mg/mL of EO treatment; ^2^ Minimal Bactericidal Concentration expressed in mg/mL of EO treatment; ^3^ Growth inhibition zone by disc diffusion assay expressed in mm; ^4^ Growth inhibition zone by vapor phase test expressed in mm. Values are expressed as means ± SD. *p* < 0.05 from one-way analysis of variance test (ANOVA).

**Table 6:** Antibacterial activity of *A. alba* EO.

Strains/Origin	*Abies alba*				
	MIC ^1^	MBC ^2^	MBC/MIC Ratio	IZ ^3^	Vapor IZ ^4^
*E. coli*	51.28	51.28	1	0.00	0.00
*P. fluorescens*	51.28	51.28	1	0.00	0.00
*A. bohemicus*	12.82	25.64	2	19.67 ± 0.58	80.00 ±00
*K. marina*	51.28	51.28	1	7.67 ± 1.15	80.00 ± 00
*B. cereus*	12.82	25.64	2	15.00 ± 2.65	66.67 ± 11.55

^1^ Minimal Inhibitory Concentration expressed in mg/mL of EO treatment; ^2^ Minimal Bactericidal Concentration expressed in mg/mL of EO treatment; ^3^ Growth inhibition zone by disc diffusion assay expressed in mm; ^4^ Growth inhibition zone by vapor phase test expressed in mm. Values are expressed as means ± SD. *p* < 0.05. *p* < 0.05 from one-way analysis of variance test (ANOVA).

**Table 7:** Effects of *P. mugo*, *P. cembra*, *P. abies*, and *A. alba* EOs in different antioxidant assays.

Assay	Values Expressed as	*P. cembra*	*P. mugo*	*P. abies*	*A. alba*
DPPH	IC_50_ *	13.01 ± 0.86	3.08 ± 0.65	13.05 ± 3.09	7.84 ± 1.70
	TEAC **	1.63 ± 0.46	7.65 ± 1.33	1.68 ± 0.64	3.01 ± 0.48
ABTS	IC_50_ *	44.90 ± 2.06	43.08 ±6.95	45.00 ± 6.26	44.23 ± 1.10
	TEAC **	13.26 ± 1.45	14.01 ± 2.01	13.26 ± 0.52	13.65 ± 0.49

* µg/mL of essential oil; ** µM of Trolox equivalent/mg of essential oil. Values are expressed as means ± SD. *p* < 0.05.

## Data Availability

Not applicable.
